# Quality and Shelf-Life Stability of Pork Meat Fillets Packaged in Multilayer Polylactide Films

**DOI:** 10.3390/foods11030426

**Published:** 2022-02-01

**Authors:** Eva Hernández-García, María Vargas, Sergio Torres-Giner

**Affiliations:** Research Institute of Food Engineering for Development (IIAD), Universitat Politècnica de Valencia (UPV), 46022 Valencia, Spain; evherga1@upvnet.upv.es (E.H.-G.); mavarco@tal.upv.es (M.V.)

**Keywords:** PLA, multilayer films, sustainable packaging, food quality and shelf life, meat preservation

## Abstract

In the present study, the effectiveness of a multilayer film of polylactide (PLA), fully bio-based and compostable, was ascertained to develop a novel sustainable packaging solution for the preservation of fresh pork meat. To this end, the multilayer PLA films were first characterized in terms of their thermal characteristics, structure, mechanical performance, permeance to water and aroma vapors and oxygen, and optical properties and, for the first time, compared with two commercial high-barrier multilayer packaging films. Thereafter, the multilayers were thermosealed to package fillets of fresh pork meat and the physicochemical changes, lipid oxidation levels, and microbiological counts were monitored in the food samples during storage under refrigeration conditions. Results showed that the meat fillets packaged in PLA developed a redder color and showed certain indications of dehydration and oxidation, being more noticeably after 11 days of storage, due to the higher water vapor and oxygen permeance values of the biopolymer multilayer. However, the pH changes and bacterial growth in the cold-stored fresh pork meat samples were minimal and very similar in the three tested multilayer films, successfully accomplishing the requirements of the food quality and safety standards at the end of storage.

## 1. Introduction

Food preservation aims to extend shelf life and provide safer products to consumers using different materials and technologies [1]. Advances in packaging materials have played an essential role in food preservation [2]. In the last decade, plastics, that is, polymers and additives, have extensively contributed to food preservation due to their balanced properties (e.g., transparency, flexibility, low cost, ease of processing, low weight, etc.) and high versatility based on the wide variety of formulations for specific product requirements [3]. In addition, food packaging can work beyond conventional protective properties, providing other functions to the food product, such as active and bioactive properties, convenience, and communication [4].

Barrier developments in the food packaging area have greatly helped to reduce food waste since the quality of most food products deteriorates due to mass transfer phenomena, such as moisture adsorption/desorption, the invasion of oxygen, flavor loss as well as sorption of undesirable odors, or the migration of the components from the container to the food. However, barrier packaging developments are not based on a single polymer since, in most cases, it does not fully meet the permeability requirements necessary for all vapors and gases [5]. Thus, to achieve the high-barrier performance required for correct food preservation, in addition to minimizing material costs and achieving lidding film adhesion, the food packaging industry primarily employs structures composed of multiple polymer layers. The so-called multilayer structures usually consist of up to 12 or even more layers of different polymers and/or coatings, in either symmetrical or asymmetrical assemblies, specially designed to be impervious to the penetration and migration of gases and moisture [6]. Nevertheless, multilayer packaging materials have created severe problems related to the disposal of plastics after single short use due to the polymers used are non-biodegradable and their layers are extremely difficult to separate and recycle [7]. In fact, in developed countries, an important part of municipal solid waste consists of polymer materials derived from multilayer structures [8]. Therefore, the development of sustainable solutions and management of post-consumer plastics represents a fundamental challenge in the food packaging area.

Innovations in sustainable food packaging appear as a way to minimize the environmental problems of petrochemical polymers. In this scenario, biopolymers offer the advantages of being produced from renewable resources and/or reintegrated into the carbon cycle through biodegradation by microorganisms and enzymes in a natural environment or compost [9,10]. Among biopolymers, polylactide (PLA) is a linear aliphatic polyester obtained from carbohydrate-rich plants, such as corn or wheat. Starch is separated and converted to dextrose (_D_-glucose) through enzymatic hydrolysis and fermented to lactic acid [11]. PLA is, thereafter, industrially synthesized by ring-opening polymerization (ROP) of lactide, the cyclic diester formed from two molecules of lactic acid with loss of two molecules of water, to achieve high molecular weights (M_W_s) without solvents [12]. The resultant PLA packaging articles can be biodisintegrated with the right temperature and humidity conditions in industrial composting facilities, offering a sustainable alternative to non-biodegradable petroleum derived polymers [13]. PLA exhibits similar mechanical strength and higher transparency than polyethylene terephthalate (PET) or polystyrene (PS) [14]. Thus, PLA is ideal for rigid and transparent film applications and it can also be used in food trays, bottles, candy wraps, and cups [15]. Currently, various types of foods are packaged in PLA-based materials, with a high range of physical properties, water activity, and pH, such as fresh vegetables and salads [16]. However, the use of PLA films has been scarcely explored in meat preservation.

Pork and beef meat products play an important role in worldwide nutrition with large increases in global consumption due to key micronutrients and protein content. However, fresh red meat is highly perishable due to its biological composition and its shelf life is dependent on pre-slaughter, processing, and post-processing conditions [17]. Meat deterioration could lead to the decrease in the nutritional value and changes in appearance and production of off-flavors and odors. Microbial spoilage, water exudates, color loss due to myoglobin oxidation, and lipid oxidation represent the first indicators of meat deterioration [18]. With the increased demand for high-quality, safe, and extended shelf life of fresh products, multilayer packaging is commonly used in the meat industry. For instance, fresh pork can be only preserved for six days upon hygienic and temperature control, whereas its shelf life can be extended for up to 3 weeks when stored in vacuum packaging (VP) or modified atmosphere packaging (MAP, 0.4% carbon monoxide, ~60% carbon dioxide, and ~40% nitrogen) [19]. Although the presence of oxygen can be beneficial during the retail display to provide a bright red color that consumers associate with freshness, completely anoxic environments optimize keeping quality.

The present study aims to evaluate the physical properties, in terms of thermal, mechanical, barrier, and optical properties of multilayer PLA films and their feasibility for preserving fresh pork meat. To this end, the quality and shelf life of the meat packaged in the PLA films were compared with that preserved in two commercial multilayer films with a high barrier that are habitually used for meat packaging.

## 2. Materials and Methods

### 2.1. Materials

A bioriented PLA (BOPLA) film of 20 µm and labeled as compostable according to the “OK bio-based” (S206) and DIN EN 13432 (7H0052) standards, respectively, was provided as NATIVIA^®^ NTSS 20 by Taghleef Industries S.L. (Jaén, Spain). It is a multilayer film composed of three layers of PLA that is heat-sealable on both sides, where the external layers have been modified to confer a minimum sealing temperature (MST) of 85 °C. A high-barrier multilayer film based on polyamide 6 (PA6) and poly(ethylene-*co*-vinyl alcohol) (EVOH), with a total thickness of 140 µm, was supplied as WK140 by WK THOMAS (Barcelona, Spain). A multilayer film, also based on EVOH and with a total thickness of 100 µm, was obtained from Cryovac Inc. (Sealed Air Spain, Buñol, Spain) with commercial reference VST200P. These commercial multilayers were marked as PLA, M1, and M2, respectively. The commercial multilayers M1 and M2 have been designed by manufacturers for lamination applications in barrier packaging, including the preservation of meat. A commercial polyvinyl chloride (PVC) cling film (Bosque Verde, Mercadona S.A., Valencia, Spain), employed for food wrapping applications, was also used as a control to handle and protect the non-packaged samples.

Absolute ethanol (EtOH) and _D_-limonene were both supplied by Sigma-Aldrich S.A. (Madrid, Spain). Magnesium nitrate-6-hydrate [Mg(NO_3_)_2_], trichloroacetic acid (TCA), and thiobarbituric acid (TBA) reagent were all supplied by Panreac Química, SA (Castellar del Vallés, Spain).

The pork meat was purchased in the form of a single piece of 1 kg from the loin from a local supermarket (Consum S. Coop. V., Valencia, Spain). The food samples were immediately transported to the laboratory facilities in a portable fridge at 5 °C and processed into fillets using a professional slicer (Smarty 250 IX, Manconi, Italy) in a cabinet. Microbiological media, that is, buffered peptone water, Violet Bile Red agar (VRB), and Plate Count agar (PCA) were provided by Scharlab S.L. (Barcelona, Spain). Man Rogosa and Sharpe Agar (MRS) were obtained from Lankem-Labbox (Barcelona, Spain).

### 2.2. Film Characterization

#### 2.2.1. Thickness and Conditioning

Prior to characterization, the whole thickness of all films was measured using an electronic digital micrometer (Comecta S.A., Barcelona, Spain), having ±0.001 mm accuracy. Measurements were performed at six random points and values were averaged. Thereafter, the samples were preconditioned at 25 °C and 53% relative humidity (RH) for 1 week.

#### 2.2.2. Thermal Characterization

Differential scanning calorimetry (DSC) was carried out to determine the melting temperature (T_m_) of the polymers present in the multilayers using a DSC823^℮^ Star^℮^ (Mettler-Toledo GmbH, Schwerzenbach, Switzerland). Films samples (~10 mg) were placed into aluminum pans, tightly sealed, and heated from −50 °C to 250 °C at 10 °C/min. As a reference, an empty aluminum pan was used.

Their thermal degradation was analyzed by thermogravimetric analysis (TGA) using a Star^℮^ System analyzer (Mettler-Toledo GmbH). Film samples (~3 mg) were subjected to a heating program from 25 °C to 600 °C at a heating rate of 10 °C/min under a nitrogen atmosphere (10 mL/min). In each test, the corresponding mass curve was obtained as a function of temperature. All thermal tests were performed in triplicate.

#### 2.2.3. Multilayer Structure Determination

The determination of the number of layers and each layer thickness was performed by image analysis using light optical microscopy (Olympus BX50 light microscope, Tokyo, Japan) using 50× magnification. The samples were cross-sectioned with a microtone (Microtone M240, Especialidades Médicas Myr, S.L., Tarragona, Spain) and stained with an iodopovine solution (10 g/100 mL, Viatris Inc., Madrid, Spain).

The microstructure and surface of the films were observed by Field Emission Scanning Electron Microscope (FESEM, Ultra 55, Zeiss, Oxford Instruments, Abingdon, UK). To this end, the samples were mounted on holders using double-sided carbon tape, coated with a platinum layer (EM MED020 sputter coater, Leica Biosystems, Barcelona, Spain), and observed using an accelerated voltage of 2 kV. The film samples were previously cryo-fractured with liquid nitrogen to obtain their cross-sections.

#### 2.2.4. Mechanical Analysis

The mechanical properties of the multilayer films were evaluated using a universal mechanical testing press (Stable Micro System TA-XT plus, Haslemere, England), following the standard method ASTM D882 [20]. The samples, sizing 25 mm × 100 mm, were positioned in tension test clips (Model A/TG, Stable Micro Systems) and subjected to a tensile test at a speed of 50 mm/min until breaking. The force–distance curves obtained in the test were transformed into Henky stress–strain curves.

#### 2.2.5. Permeance Measurements

Water vapor permeance of the multilayer films was determined according to ASTM E96/E96M gravimetric methodology [21] at 25 °C and 53% RH. Film samples (Ø = 3.5 cm) were placed and sealed in Payne permeability cups filled with 5 mL of distilled water (100% RH). Then, the cups with the films were placed into desiccators containing an Mg(NO_3_)_2_ over-saturated solution and weighed periodically (ME36S, ± 0.00001 g accuracy, Sartorius, Goettingen, Germany) for one week. The water vapor permeance was calculated considering the water vapor transmission rate (WVTR), which was determined from the slope of the weight loss vs. time and correcting for permeant partial pressure. For the permeance to limonene, the procedure was similar to that described previously for water vapor, placing 5 mL of _D_-limonene inside the Payne permeability cups and storing them under the controlled environmental conditions of 25 °C and 53% RH. Limonene permeation rate (LPR) was obtained from the steady-state permeation slopes of weight loss vs. time and corrected for permeant partial pressure. In both cases, cups with aluminum films were used as control samples to estimate and subtract the vapor loss through the sealing. All the vapor permeance measurements were performed in triplicate.

The oxygen permeance of the films was determined using an Ox-Tran equipment Model 1/50 (Mocon, Minneapolis, MN, USA) according to ASTM D3985-05 [22]. Tests conditions were also set at 25 °C and 53% RH and the exposure area during the test was 50 cm^2^. The permeance values were derived from the oxygen transmission rate (OTR) measurements, which were corrected with the gas partial pressure and recorded in triplicate.

#### 2.2.6. Optical Evaluation

The optical properties of the multilayer films were determined, in triplicate, by measuring the reflection spectrum of the films in the wavelength range from 400 to 700 nm using a MINOLTA spectro-colorimeter (model CM-5, Minolta Co., Tokyo, Japan). Transparency was measured by the internal transmittance (*Ti*) at 700 nm, applying the Kubelka–Munk theory of multiple scattering to determine the film reflection spectra (*R*) using the black (*R*_0_) and white (*R*_g_) backgrounds, whereas the CIE L*a* b* (CIELAB) color coordinates were determined using Equations (1)–(3), considering the illuminant D65 and 10° observer:

(1) $$\;T_{i}=\sqrt{{\left(a+R_{0}\right)}^{2}-b^{2}}$$

(2) $$a=\frac{1}{2}\;\left[R+\left(\frac{R_{0}-R+R_{g}}{R_{0}\times R_{g}}\right)\right]\;\;\;\;\;\;$$

(3) $$b=\sqrt{a^{2}-1}$$

The chromatic parameters hue angle (h_ab_*), chroma (C_ab_*), and opacity (*O*) were obtained from Equations (4)–(6), respectively:

(4) $$h_{ab}{}^{*}=arctg\left(\frac{b^{*}}{a^{*}}\right)$$

(5) $$C_{ab}{}^{*}=\sqrt{a^{*2}+b^{*2}}$$

(6) $$O=A_{500}\times L$$

where *A*_500_ and *L* correspond to the absorbance at 500 nm and the film thickness, respectively.

### 2.3. Pork Meat Characterization

#### 2.3.1. Preparation of Pork Meat Samples

Prior to preparing the samples, all utensils and work surfaces were disinfected to avoid cross-contamination with 96% ethanol (Panreac SA, Barcelona, Spain). The as-received multilayer films were also sterilized by exposure to ultraviolet (UV) light for 30 min in a laminar flow cabinet (Bio II Advance, Telstar, Terrassa, Spain). Then, the pork meat was processed into 10 g slices using a professional slicer (Smarty 250 IX, Manconi, Italy) and immediately placed inside the multilayer films. To this end, the films were first cut into samples, sizing 10 cm × 10 cm, and heat-sealed with a vacuum sealer machine (Vacio Press Elite, SAECO, Gaggio Montano, Italy), placing the meat fillets in between. Some pork meat fillets were also wrapped in PVC cling films to be used as the control samples (without packaging).

All samples were stored under refrigerated conditions, at 5 °C and 48% RH, for up to 15 days to carry out the evaluation. These conditions were selected according to previous studies that evaluated the performance of biodegradable packaging films for meat preservation applications [23,24].

#### 2.3.2. Physico-Chemical Evaluation

The pH values were determined using a digital pH meter by direct insertion of the electrode probe (Mettler-Toledo GmbH) into the pork meat samples. The following measurements were performed at day 0 (unpackaged) and at days 3, 7, 11, and 15. Five measurements were taken, in duplicate, for each sample.

Weight loss of the samples was quantified as a function of storage time with a scale (ME36S, ±0.00001 g accuracy) from Sartorius. The optical properties of the packaged pork meat were also characterized by measuring the reflection spectrum of the packaged samples at a wavelength of 400 to 700 nm at six random points on the sample surface, using the MINOLTA colorimeter spectrum, with a standard white plate as a background. Color coordinates (CIE L*a* b*), hue (h_ab_*), chroma (C_ab_*), and total color difference (Δ*E*, Equation (7)) were measured during storage using the illuminant D65/10° observer.

(7) $$\Delta E=\sqrt{{\left(\Delta L^{*}\right)}^{2}+{\left(\Delta a^{*}\right)}^{2}+{\left(\Delta b^{*}\right)}^{2}}$$

The degree of lipid oxidation was evaluated by quantifying the thiobarbituric acid reactive species (TBARS) according to the method described by Siu and Draper [25]. For this, at the beginning and end of storage, 10 g of each sample were placed in bags (Stomacher 440 Classic Strainer Bags, Worthing, UK) with 50 mL of distilled water and homogenized for 2 min using a homogenizer (Masticator Paddle blender, IUL Instruments, Barcelona, Spain). Then, 50 mL of 10 (vol/vol%) TCA was added and the homogenate was filtered with a vacuum pump using Whatman #1 filter paper (Whatman Nº1, Whatman International Ltd., Kent, UK). Thereafter, 8 mL of the clear filtrate was added to 2 mL of 0.06 M TBA reagent and incubated for 90 min at 80 °C. The absorbance was read at 532 nm and the results were expressed as mg of malonaldehyde (MDA) per kg of meat.

#### 2.3.3. Microbial Analysis

Pork samples were analyzed for bacterial growth at the selected different storage times, that is, 0, 3, 7, 11, and 15 days. A total of 10 g of meat sample packaged in each of the multilayer films was aseptically taken using sterile tweezers in the laminar flow cabinet and, subsequently, placed in sterile bags (Stomacher 440 Classic Strainer Bags) with 90 mL of peptone water (Scharlab S.L.). The Stomacher bags were homogenized for 3 min using a homogenizer (Masticator Paddle blender, IUL Instruments). Thereafter, serial decimal dilutions were prepared and plated on PCA, VRB, and MRS agars for the total aerobic counts (TAC), total coliforms, and lactic acid bacteria (LAB) counts, respectively. The plates used for the total coliform and total aerobic counts were incubated at 37 °C for 48 h, while the conditions for those used for the LAB counts were 30 °C for 72 h. After incubation, all the colonies were counted, and the results were expressed as colony-forming units per gram (CFU/g). All the microbial tests were performed in triplicate.

### 2.4. Statistical Analysis

Results were subjected to analysis of variance (ANOVA) using Statgraphics Centurion XVII-64 software (Manugistics Corp., Rockville, MD, USA). To this end, significant differences were assumed with a significance level greater than 95% (*p* < 0.05).

## 3. Results and Discussion

### 3.1. Multilayer Structure

**Figure 1:**
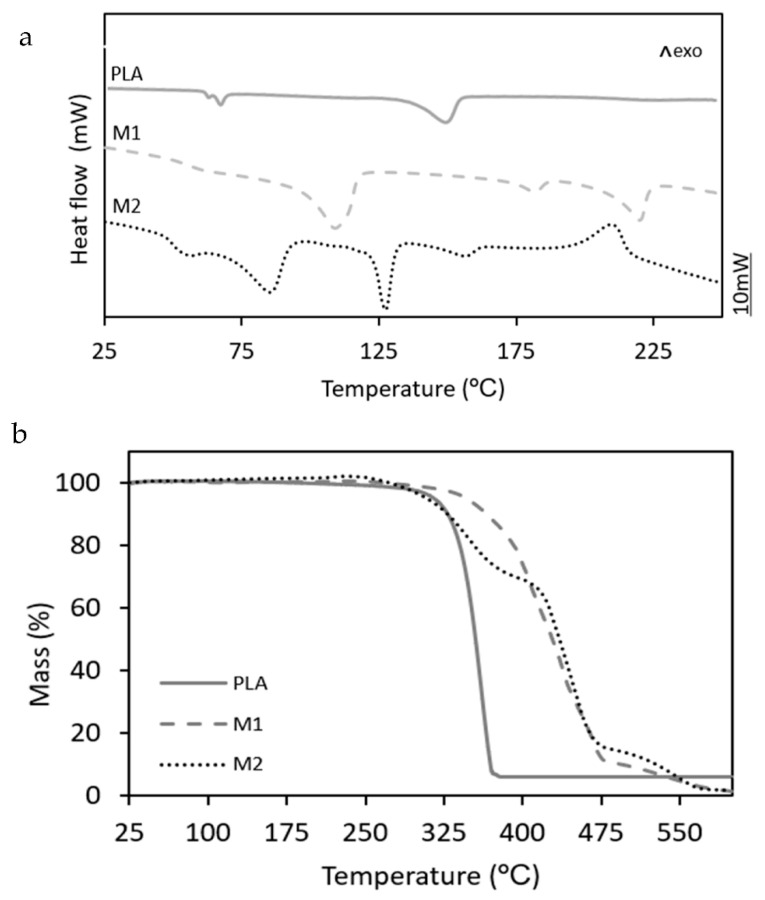
(**a**) Differential scanning calorimetry (DSC) thermograms and (**b**) thermogravimetric analysis (TGA) curves of the polylactide (PLA), M1, and M2 multilayer films.

Prior to evaluating the performance of the multilayers, their structures were ascertained by determining the thermal properties and observing the film samples in the microscope. In Figure 1, the DSC and TGA curves of the three multilayer films are gathered. One can observe in Figure 1a, showing the DSC thermograms, that the PLA multilayer showed a glass transition temperature (T_g_) at ~67 °C, having a low-intensity endothermic peak due to physical aging or the presence of a plasticizer with a low-melting profile, followed by a T_m_ value of 148.9 °C. These thermal transition values are characteristic of PLA, confirming the absence of other semicrystalline polymers in the film sample. The M1 multilayer showed three endothermic peaks, centered at 109.8 °C, 182.4 °C, and 220.3 °C, which can be respectively ascribed to the melting process of low-density polyethylene (LDPE), poly(ethylene-*co*-vinyl alcohol) with 32 mol% ethylene content (EVOH32), and PA6. In the case of M2, this multilayer film sample also yielded multiple endothermic peaks, particularly observed at 56.4 °C, 85.8 °C, 127.3 °C, and 156.4 °C. The first two melting peaks suggest the presence of a metallocene polyethylene wax with a very low melting point and a copolymer of ethylvinylacetate (EVA). The latter copolymer combines molecules of a hydrophilic and a hydrophobic part, which leads to an excellent tie-layer behavior [26]. The medium melting point can be ascribed to high-density polyethylene (HDPE) or a propylene-ethylene copolymer (coPP). The following low-intensity exothermic peak can be related to the melting of poly(ethylene-*co*-vinyl alcohol) with 44 mol% or, more probably, 48 mol% ethylene contents (EVOH44 or EVOH48) [27]. Finally, the last and intense exothermic peak observed at ~200 °C can be due to the deacetylation of EVA. In particular, copolymers with high vinyl acetate contents (up to 50 wt%) can release acetic acid prior to thermal decomposing at higher temperatures [28].

Figure 1b shows the TGA curves of the different multilayer films to better ascertain their composition and corroborate the DSC results shown above. From these curves, the corresponding values of T_onset_ (initial degradation temperature) and T_deg_ (temperature of maximum degradation rate) were determined. In the case of PLA, one can observe that the film degraded in a single and rapid step, showing values of T_onset_ and T_deg_ of 310.6 °C and 360.3 °C, respectively. These values are similar to those reported previously for PLA, which releases lactic acid, oligomers of lactic acid (OLAs), acetaldehydes, carbon dioxide, carbon monoxide, and ketones during thermal decomposition [29]. A similar degradation profile, but with higher thermal stability, was observed in the case of the M1 film. In particular, this commercial multilayer sample was thermally stable up to 350.4 °C and then degraded in two steps with T_deg_ values of 444 °C and 546.4 °C. The presence of two degradation peaks has been reported for the thermal degradation of polyolefins, which is based on the decomposition of the C–C covalent bonds followed by the breakdown, at a lower decomposition rate, of the polymer chains by free radicals [30]. Finally, the M2 sample presented the typical thermal degradation profile of EVA, showing an initial mass loss from 180.2 °C that involves the formation and release of acetic acid, the so-called deacetylation, being more noticeable in the 325–410 °C range, followed by degradation of the ethylene blocks [28]. For EVOH, T_deg_ values of approximately 391 °C and 409 °C have been reported for EVOH32 and EVOH44, respectively, which are also in the decomposition range observed for the multilayers studied herein [31].

**Figure 2:**
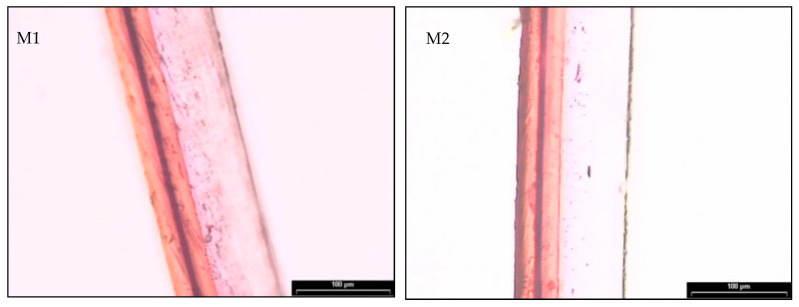
Stained cross-sections of the M1 (**left**) and M2 (**right**) multilayer films.

Layer thickness and distribution of the commercial multilayers were determined by light optical microscopy after staining. Figure 2 shows the cross-section of the M1 and M2 multilayers, where one can observe the presence of different layers by having different color contrasts. In the M1 multilayer sample, up to 4 layers can be seen. The thicker one of approximately 90 µm, with lower color contrast, can be ascribed to LDPE. Then, three colored layers can be identified, having a different contrast. In the case of EVOH, the staining was more intense due to the higher number of hydroxyl groups, which correspond to the inner layer with a thickness of 5 µm. This layer was surrounded by two PA6 layers, having a thickness of approximately 20 µm. The morphological analysis of the M2 indicated the presence of 5 layers. The structural one, without color and of approximately 60 µm, that corresponds to the polyolefin. Then, the layers stained can be ascribed to two thin layers EVOH of 5 µm, the most intense ones, that adhered to two layers of EVA, of approximately 16 µm. The presence of EVA, as suggested above during the DSC and TGA evaluations, would contribute to improving layer adhesion and toughness in the multilayer, while EVOH is aimed to provide barrier to oxygen, odors, and flavors.

**Figure 3:**
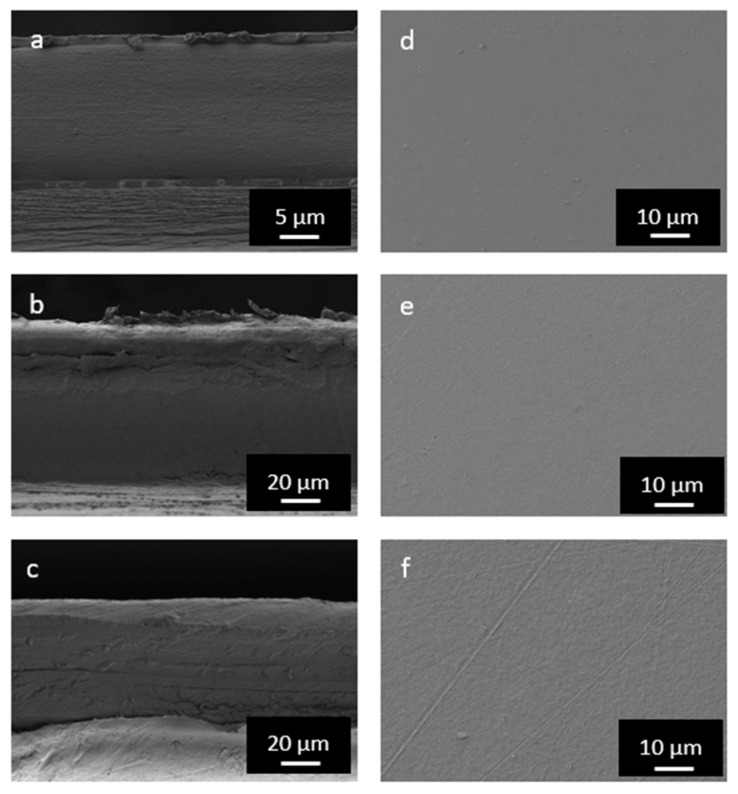
Field emission electron microscopy (FESEM) micrographs of the polylactide (PLA) (**a**,**d**), M1 (**b**,**e**), and M2 (**c**,**f**) multilayer films in their cross-sections (left) and top views (right).

The structure of the multilayers was further analyzed and confirmed by FESEM. Figure 3 shows the cross-sectional (left images) and superficial micrographs (right images) of the film samples. One can see that the PLA film was composed of 3 layers, having the external thermosealable layers a mean thickness of approximately 1 µm. In the case of the M1, it can be discerned that this film sample was composed of 4 layers since the EVOH layer started to delaminate from the surrounding PA6 layers due to the cryo-fracture process. Finally, the presence of several layers can be inferred in the M2 film, where the thickest layer, shown at the top, would correspond to the polyolefin. Moreover, the top views revealed that all multilayer films exhibited homogeneous and smooth surfaces without any pores. Similar morphologies were previously reported for multilayer film structures based on PLA [32]. Table 1 summarizes the layer thickness obtained from the microscopy analysis.

Therefore, from the structural analysis, one can consider that the PLA film was composed of a three-layer structure of the neat biopolymer, whereas the two commercial multilayers presented a structure of PA6/EVOH32/PA6/LDPE (M1) and EVOH48/EVA/EVOH48/EVA/coPP (M2). Although EVOH is a non-chlorine barrier material for fresh meat packaging, copolymers with lower ethylene contents and, hence, higher oxygen barrier, are very sensitive to moisture conditions, especially at a relative humidity of above 80%. Therefore, as in the case of M1, this copolymer, EVOH32, was incorporated in multilayer structures protected between water-barrier polymers. For M2, EVOH48 was applied in the external layer due to its higher ethylene content. In both cases, sustainable alternatives are urged due to the increasing environmental concerns related to its poor recyclability.

### 3.2. Mechanical Properties of Multilayers

**Figure 4:**
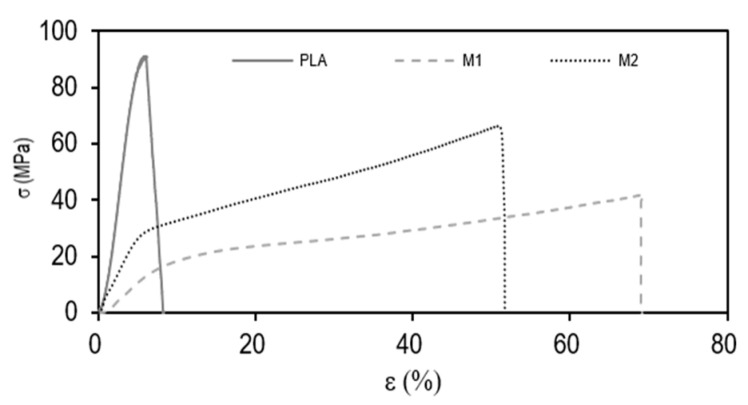
Typical stress (σ) versus deformation (ɛ) curves of the polylactide (PLA), M1, and M2 multilayer films.

Figure 4 shows the tensile stress–strain curves of the multilayer films that allowed obtaining the parameters of elastic modulus (E), tensile stress at break (σ_b_), and deformation at break (ɛ_b_). One can observe that the three tested films presented a very dissimilar mechanical performance, suggesting different applications. On the one hand, the M1 multilayer presented the lowest mechanical resistance, yielding values of E and σ_b_ of 258 ± 24 and 42 ± 3 MPa, respectively, and ɛ_b_ of 67 ± 8%. These values correspond to a flexible film with high ductility. In contrast, the multilayer PLA film showed E and σ_b_ values of 2167 ± 209 and 89 ± 4 MPa, respectively, with a ɛ_b_ value of 5 ± 1%. This mechanical performance is related to strong but brittle materials. In the case of M2, this multilayer presented intermediate values but closer to those of the M1 multilayer, that is, E, σ_b_, and ɛ_b_ values of 529 ± 27 MPa, 71 ± 9 MPa, and 56 ± 8%, respectively. Therefore, in terms of mechanical performance, the commercial multilayer films of PA6/EVOH32/PA6/LDPE and EVOH48/EVA/EVOH48/EVA/coPP presented characteristics of films suitable for flexible packaging applications, while the PLA multilayer film, being more rigid and less deformable, can be of more interest for rigid packaging uses such as trays, lids, or protective sheets. It is also worth indicating that the mechanical properties attained for the PLA films are similar to those reported for both injection-molded parts [33] and thermo-compressed films [34] made of neat PLA. Thus, it can be considered that the presence of the external thermosealable PLA layers did not alter the original mechanical characteristics of the biopolyester, and good adhesion between these with the inner PLA layer can be inferred due to they share the same composition.

### 3.3. Barrier Properties of Multilayers

Table 2 shows the thicknesses, measured by the micrometer, and barrier properties to water and limonene vapors and oxygen of the multilayer films. Barrier performance was expressed in terms of permeance since it is determined not only by the intrinsic permeability of each constituent material of the multilayer structure but also by thickness sample. As it can be seen in the table, all film samples showed significant differences (*p* < 0.05) and the M1 multilayer presented the highest barrier to water vapor and oxygen gas. In particular, the values of permeance to the vapors of water and limonene were 5.79 × 10^−12^ and 1.51 × 10^−10^ kg.m^−2^.Pa^−1^.s^−1^, respectively, whereas the oxygen permeance showed a value of 2.15 × 10^−16^ m^3^.m^−2^.Pa^−1^.s^−1^. This high-barrier performance can be related to both the low permeability of its constituents and the higher film thickness, that is, nearly 140 µm. In particular, the LDPE polyolefin contributes to its high-water vapor barrier since it shows a very low water vapor permeability with a value of 1.2 × 10^−15^ kg.m.m^−2^.Pa^−1^.s^−1^ at 38 °C and 90% RH (standard tropical conditions) [35]. It is worth mentioning that the equivalent water vapor permeance of a 137-µm film made of neat LDPE results in a value of 8.76 × 10^−12^ kg.m^−2^.Pa^−1^.s^−1^, which is in the order but nearly two times higher than that of the multilayer film sample tested herein. This difference could be attributed to the lower temperature used herein to determine the barrier properties, that is, 25 °C, or potential treatment of the film with a water-barrier coating, such as silicon oxide (SiOx) (1.3 × 10^−17^ kg.m.m^−2^.Pa^−1^.s^−1^ at 38 °C and 90% RH) [36]. In terms of the oxygen barrier, the presence of PA6 and, more importantly, EVOH32 can contribute to its very low permeance, particularly at low humidity conditions that are achieved when these polymers are protected by LDPE at the external layers. For instance, in the case of EVOH32, that is, the copolymer containing 32 mol% of ethylene, oxygen permeability varies from 0.77 to 91 × 10^−21^ m^3^.m.m^−2^.Pa^−1^.s^−1^ for 0 and 75% RH, respectively, at 23 °C [35]. One can further observe that the M2 sample, that is, EVOH48/EVA/EVOH48/EVA/coPP multilayer, also presented a high-barrier performance, but still significantly lower (*p* < 0.05) than that of PA6/EVOH32/PA6/LDPE in terms of water vapor and oxygen. This multilayer showed permeance values of 2.32 × 10^−11^ kg.m^−2^.Pa^−1^.s^−1^ and 9.71 × 10^−15^ m^3^.m^−2^.Pa^−1^.s^−1^ for water vapor and oxygen, respectively. In comparison to the other commercial multilayer, this lower permeance can result from its lower thickness, that is, 98 µm, and also, in the case of oxygen, due to its higher permeability since it is based on an EVOH copolymer with a lower vinyl alcohol content. For instance, EVOH44 is nearly 6 times more permeable to oxygen than EVOH32 [27]. It is remarkable to note, however, that the limonene permeance of the M2 multilayer was lower than that of M1, with respective values of 6.94 × 10^−11^ and 1.51 × 10^−10^ kg.m^−2^.Pa^−1^.s^−1^, suggesting that the aroma barrier of EVOH increases at higher ethylene contents.

In the case of the PLA film, it can be observed that its permeance to water vapor was about 20–200 times higher than that of the two commercial multilayer films, while it was in the same order of magnitude in terms of limonene vapor. This 20-µm PLA multilayer yielded permeance values of 9.26 and 2.20 × 10^−10^ kg.m^−2^.Pa^−1^.s^−1^ for water and limonene vapors, respectively. These vapor permeance values result in equivalent permeability values for a monolayer of PLA of 1.85 × 10^−14^ and 4.42 × 10^−15^ kg.m.m^−2^.Pa^−1^.s^−1^, respectively, which are very similar to those reported in the literature for PLA (1.23 × 10^−14^ and 3.30 × 10^−15^ kg.m.m^−2^.Pa^−1^.s^−1^ at 25 °C) [37], confirming that the multilayer was fully based on PLA. The slight reductions observed in the vapor barrier properties in comparison with neat PLA could be ascribed to the plasticization performed on the outer layers to yield a film with enhanced thermosealability. In comparison with its petrochemical counterpart, PET, one can consider that the PLA film yielded lower vapor barrier performance to water but higher to limonene. This is based on the fact that according to the vapor permeabilities values of PET reported in the literature [38], an equivalent 20-µm PET film would provide permeance values of 1.15 × 10^−10^ and 5.85 × 10^−9^ kg.m^−2^.Pa^−1^.s^−1^ for water and limonene, respectively. In relation to the oxygen permeance, the PLA film resulted in a value of 1.15 × 10^−13^ m^3^.m^−2^.Pa^−1^.s^−1^, which was 2.5 and 1 orders of magnitude higher than in the M1 and M2 multilayer films, respectively. The resultant permeability of the PLA film assuming a monolayer is 2.31 × 10^−18^ m^3^.m.m^−2^.Pa^−1^.s^−1^, being very similar to that reported recently for PLA (2.22 × 10^−18^ m^3^.m.m^−2^.Pa^−1^.s^−1^ at 25 °C and 60% RH) [34] and also 10–15 times higher than that of PET (3.27 and 4.26 × 10^−19^ m^3^.m.m^−2^.Pa^−1^.s^−1^ at 23 °C and 0% and 75% RH, respectively) [38]. Therefore, the present results confirm that the PLA biopolymer is an intermediate barrier material for vapors but with a low-to-intermediate barrier to oxygen [39]. According to these results, the PLA multilayer film showed significantly higher permeance values to water vapor and, more significantly, oxygen than the commercial multilayers. However, the aroma permeance of the three multilayer films was in the same range, even though the PLA film thickness was considerably lower.

### 3.4. Optical Properties of Multilayers

The optical properties of the films are shown in Table 3. One can observe that the M1 and M2 multilayer samples, that is, PA6/EVOH32/PA6/LDPE and EVOH48/EVA/EVOH48/EVA/coPP films, showed very similar values for luminance (L*), in the 84–86 range, while the PLA multilayer presented a significantly (*p* < 0.05) higher value, that is, 94.9. As for chroma (C_ab_*), the PLA film also showed the highest value (4.3), followed by the M1 (2.6) and M2 (1.9) multilayers. In terms of the hue values (h_ab_*), it can be observed that there was no significant difference (*p* > 0.05) between the PLA and the M1 multilayers, with values ranging from 127 to 132, whereas the M2 multilayer presented a significantly higher value (*p* < 0.05), that is 148.

In terms of the internal transmittance (*Ti*) at 550 nm, one can observe values between 0.90 and 0.93 for all the multilayer films. Therefore, all the multilayers showed similar optical properties, being the PLA film slightly but still significantly (*p* < 0.05) more transparent, which can be beneficial for food packaging applications in terms of retail display.

### 3.5. Physicochemical Properties of Packaged Pork Meat

**Figure 5:**
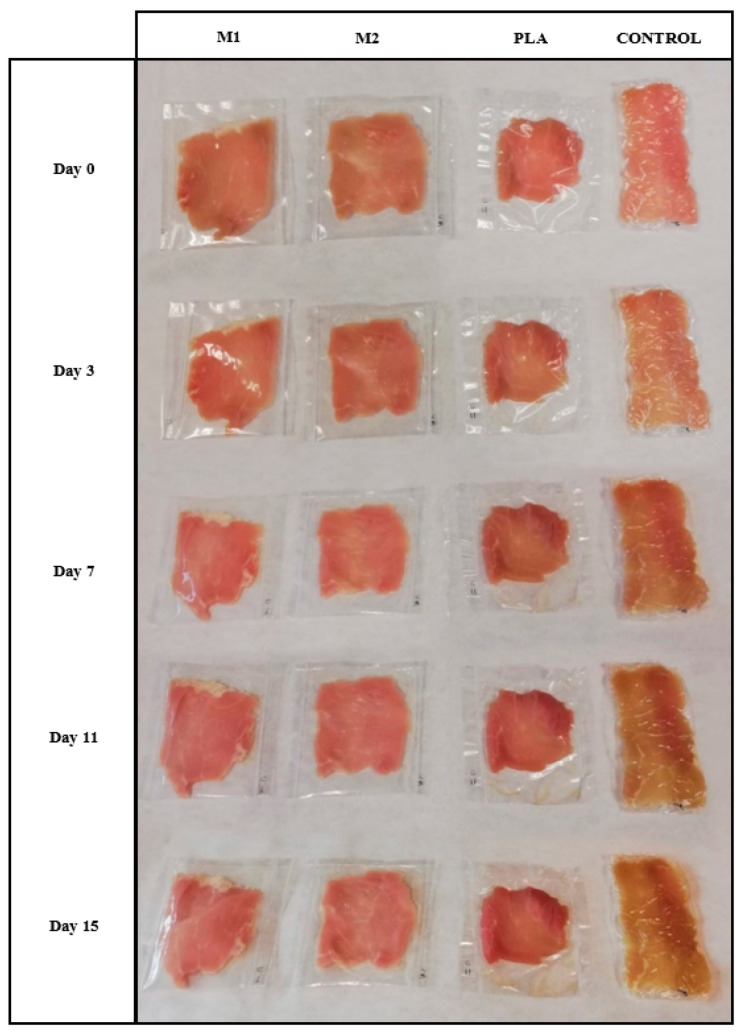
Images of pork meat fillets packaged in the polylactide (PLA), M1, and M2 multilayer films during storage.

Figure 5 shows the visual aspect of the pork meat fillets packaged in the different multilayers and control, that is, unpackaged meat wrapped in the PVC cling film during storage in refrigeration conditions of at 5 °C and 48% RH. It can be observed that the control samples suffered the most noticeable changes in color during storage, whereas the samples packaged in the PLA films developed a reddish color at the end of storage.

Table 4 shows the evolution of pH and weight loss as a function of the storage time. The initial pH of the pork meat samples was 5.49 ± 0.01, which is in the range of the values previously reported for fresh pork meat samples [40,41]. A significant effect (*p* < 0.05) of both storage time and the type of packaging was observed during the analysis of the pH values, being the type of packaging the factor having the most significant influence. During storage, it has been reported that the pH values of meat rise due to the increased content of nitrogenous bases resulting from proteolysis caused by the activity of microorganisms [42]. This behavior was clearly observed in the unpackaged meat, wrapped in PVC film, where pH increased significantly during storage up to 7.66. However, after 7 days of storage, the pH evolution of the meat packaged in the three multilayer materials was significantly different (*p* < 0.05) from that of the control meat, with pH values remaining in the 5–5.5 range. This can be explained by the air-barrier protection offered by the packaging materials, which could slow down the activity of microorganisms causing spoilage and it is also coherent with the lower oxidation level and reduced total microbial counts observed in these samples, as described below. Similar results were obtained by Daniloski et al. [43], who studied the shelf life of pork meat packaged in biaxially oriented polypropylene (BOPP) coated with acrylic/polyvinylidene chloride (BOPPAcPVDC) and biaxially oriented coextruded polypropylene (BOPPcoex) stored under vacuum in refrigeration conditions.

Furthermore, the weight loss of the pork meat during storage is also reported in Table 4. The so-called M1 and M2 multilayers, that is, PA6/EVOH32/PA6/LDPE and EVOH48/EVA/EVOH48/EVA/coPP films, resulted in a significantly lower (*p* < 0.05) weight loss during storage as compared to meat samples packaged in the PLA film. This weight variation can be related to water loss, which is consistent with the higher barrier to water vapor of the M1 and M2 multilayer films described above. This weight loss can be ascribed to the evaporation of exudate from the packaging, which is a natural event due to the leakage of intramuscular fluids from the cut surface [44]. This weight loss value could not be measured and monitored accurately in the control sample since some loss of exudate occurred during storage. Furthermore, the weight loss values attained herein are in the same range as those reported in previous studies carried out with vacuum-packaged pork chops [45] and beef steaks [46].

Lipid oxidation levels were evaluated by monitoring TBARS formation, which measures the amount of malonaldehyde (MDA) produced by secondary products of polyunsaturated fatty acid peroxidation [47]. The control samples wrapped in the PVC film showed oxidation levels of 1.90 mg MDA/kg at the end of storage. Similar values were found in other studies conducted with refrigerated pork meat at 4 °C [48]. The meat samples packaged in the multilayer films presented significantly lower (*p* < 0.05) TBARS values: 1.19, 0.77, and 0.47 mg MDA/kg for PLA, M2, and M1 films, respectively. The values obtained are consistent with the oxygen barrier properties of the materials described above. Thus, the multilayer film with the lowest oxygen permeance, that is, PA6/EVOH32/PA6/LDPE, resulted in the lowest level of lipid oxidation. In any case, one should consider that off-flavors in pork meat can generally be detected by consumers when the TBARs value is above the threshold of 0.5 mg MDA/kg [49] and that this level was surpassed at the end of storage by all samples except for those packaged in the PA6/EVOH32/PA6/LDPE films.

**Figure 6:**
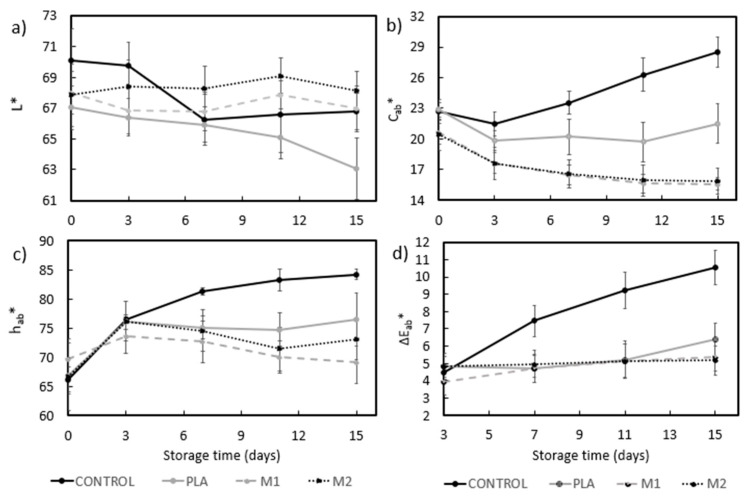
Changes in color parameters of the pork meat samples packaged in the polylactide (PLA), M1, and M2 multilayer films and wrapped in cling film (unpackaged control) during storage in terms of (**a**) Lightness (L*); (**b**) Chroma (C_ab_*); (**c**) Hue (h_ab_*); and (**d**) total color difference (ΔE_ab_*).

Figure 6 shows the evolution of the chromatic parameters (L*, C_ab_*, h_ab_*) and the total color difference, that is, Δ*E*, in relation to the initial values (t = 0). The results showed a significant effect (*p* < 0.05) of both storage time and type of packaging on the color properties of the pork meat, being the type of packaging the factor that had the greatest influence. It can be seen in Figure 6a that lightness hardly varied during the 15 days of storage in the pork meat packaged in the M1 and M2 films. In contrast, the meat packaged in the control and PLA films showed a progressive decrease with time. Among the meat samples packaged in multilayers, the highest decrease in L* detected for PLA can be explained by the higher water permeance of the biopolymer film that, in turn, is correlated with the above-mentioned largest weight loss, which occurs mainly at the food surface. Drying could yield changes in the selective light absorption on meat fillets due to changes in the refractive index of the material and also in the surface concentration of pigments that can affect the C_ab_* and h_ab_* values [50]. As described by Faustman et al. [51], oxidation of both lipids and myoglobin (Mb) in meat also leads to discoloration, and these processes are frequently linked since the oxidation of one of these compounds produces chemical species that promote the oxidation of the other. In addition, a lower pH value in pork meat has been associated with greater reflectance, which leads to an increase in lightness and a decrease in the relative amount of the reduced form of Mb. At the same time, a lower pH can be accompanied by a greater susceptibility of muscle pigments to oxygenation and oxidation. Meat yellowness increases due to an increase in the relative amounts of the oxygenated and oxidized forms of Mb, that is, oxymyoglobin and metmyoglobin (MbO_2_ and MetMb) at the expense of the reduced form [52].

In relation to the chroma or saturation (C_ab_*, see Figure 6b), it can be observed that it slightly decreased in the pork meat packaged in all the multilayer films, showing no significant effect (*p* > 0.05) between the M1 and M2 samples during storage. Samples packaged in all the three multilayer films showed a more saturated color, having no significant changes (*p* > 0.05) during storage. These differences can be mainly ascribed to the light diffraction effect of each film on the meat sample, according to their above-described optical properties. In the case of the unpackaged meat sample (control sample), as shown in Figure 6c, the h_ab_* values rose significantly (*p* < 0.05) after 11 days of storage, with a more saturated and less red hue. In contrast, the h_ab_* values of the meat samples packaged in the multilayer films slightly increased during the first 3 days of storage and, then, were kept almost constant during the remaining time of storage. It is also worth noting that the samples packaged in the PLA films developed a redder hue. These observations suggest that the unpackaged meat sample developed a more intense color due to microbial spoilage, while the food samples packaged in the multilayer films better maintained the original color of the fresh meat. In the case of PLA, the red color developed in the meat fillets can be ascribed to an oxidation process, which can be related to the lower oxygen barrier of these films. Finally, as seen in Figure 6d, for up to 11 days, the total color differences during storage of the samples packaged in the multilayer films did not exceed the usual tolerance limit for food products (Δ*E* < 5) [50], thus indicating better color preservation as compared to unpackaged samples (control).

### 3.6. Microbial Analysis of Packaged Pork Meat

**Figure 7:**
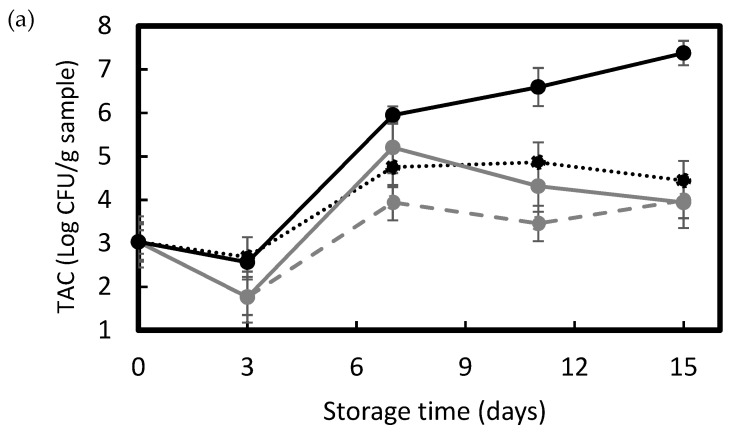

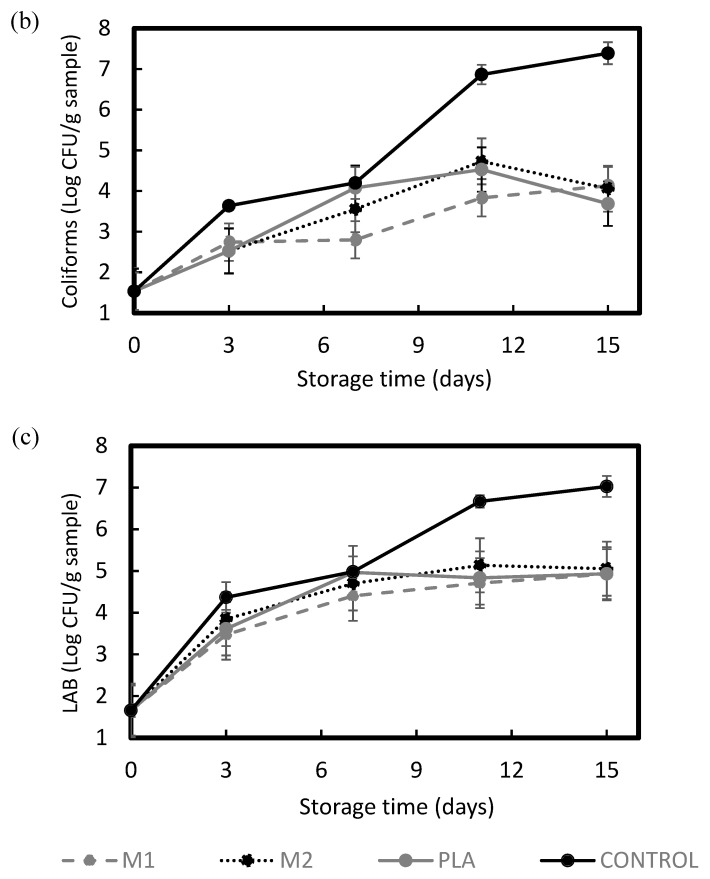
Changes in microbial counts in pork meat samples packaged in the polylactide (PLA), M1, and M2 multilayer films and wrapped in cling film (unpackaged control) during storage: (**a**) total aerobic count (TAC); (**b**) coliforms; and (**c**) lactic acid bacteria (LAB).

The main factors determining the shelf life of meat and meat products are the initial composition of the bacterial flora and the method of preservation. Among the microorganisms isolated, LAB represent the dominant group in vacuum-packaged food samples [53]. Figure 7 shows the changes in LAB counts as well as TAC and coliform counts as a function of the storage time.

The microbial analysis showed a significant effect (*p* < 0.05) of both storage time and type of packaging, being the time the factor with the greatest influence in this case. As expected, the control samples were the ones that presented higher microbial counts, though differences were only significant (*p* < 0.05) with respect to the samples packaged with the different multilayer materials after one week of storage. In terms of TAC, which is an important microbiological indicator and the quantitative standard for identifying the conditions and degree of contamination of meat [54], it can be observed that counts increased from approximately 3 log CFU/g to values in the 4.5–3.5 range for the pork meat packaged in the multilayer films during refrigerated storage (Figure 7a). The bacterial growth profile in the meat fillets was also very similar for all the multilayers, remaining nearly constant during the first days of storage and stabilizing after one week with no significant differences (*p* > 0.05) among the films. In contrast, for the unpackaged meat sample, TAC reached a value above 7 log CFU/g. In this regard, the European Commission (EC) Regulation Number 2073/2005 [54] on microbiological criteria for foodstuffs indicates that the maximum acceptable level for TAC in mechanically separated fresh pork meat is 5 log CFU/g [55]. Therefore, this level was not exceeded in the pork meat samples packaged in the multilayers for the whole studied period, accomplishing the requested value of food quality and safety.

Figure 7b shows the total coliform microbial counts, which increased from approximately 1.5 log CFU/g to values nearly 4 log CFU/g in the pork meat samples packages in the multilayer films. It can be observed that microbial counts showed very similar values, showing no significant differences (*p* > 0.05) for the three multilayers during the whole storage period. In the case of the unpackaged meat sample (control), one can observe that the total coliform counts rapidly increased after one week of storage and reached significantly higher values (*p* < 0.05) as compared to samples packaged with the multilayer films. This result correlates to the reported trend in terms of pH shown above. Finally, Figure 7c shows the microbial counts for LAB, which are the dominant group of microorganisms isolated from meat and vacuum-packaged meat products [48]. Results showed that bacterial counts increased in the meat packaged in the multilayer films from 1.3 log CFU/g to values in the 4–5 log CFU/g range after 1 week of storage. Then, bacterial counts remained almost constant during the whole storage period. As reported for the TAC and coliform counts, the growth pattern was very similar in all the pork meat fillets packaged in the multilayers, and no significant differences (*p* > 0.05) were observed in terms of the type of film. In the case of the control sample (unpackaged meat), LAB count progressively increased during storage, being significantly higher than the values reported in packaged samples after one week of storage.

Therefore, the PLA multilayer packaging proved to be effective in terms of preserving the microbiological quality of pork during storage at 5 °C, even though this film showed lower thickness and higher water vapor and oxygen permeances than the commercial multilayers based on high-barrier EVOH materials. This suggests that its medium-barrier to water and aroma can be sufficient to limit and delay microbial growth, whereas its relative low oxygen permeance can also contribute to reducing microbial counts.

## 4. Conclusions

The negative impact of multilayer food packaging materials on the environment makes it necessary to replace them with biopolymers that are derived from renewable resources and biodegradable. The properties and environmental issues of biopolymers have been widely studied during the last decade but evaluations regarding their actual performance for food preservation applications are scarce. In the present study, PLA multilayer films were thermosealed and applied, for the first time, to package pork meat fillets. The quality and shelf life of the packaged meat were determined and compared to high-barrier commercial multilayer films of PA6/EVOH32/PA6/LDPE and EVOH48/EVA/EVOH48/EVA/coPP. It was concluded that the PLA films exhibited mechanical properties typical of materials used in rigid food packaging, having a moderate barrier to water and aroma vapors and relatively low to oxygen. During the shelf-life evaluation, the PLA packaging led to slight color variations as well as certain dehydration and oxidation phenomena in the cold-stored fresh pork meat samples, particularly after 11 days of storage. However, the pH changes were minimal and similar to those observed in the food samples packaged with the commercial high-barrier multilayer films. In terms of microbial analysis, the PLA film also yielded a comparable performance to the high-barrier multilayers, and the biopolymer films accomplished the requested values of food quality and safety. Therefore, PLA can be regarded as a sustainable alternative packaging material for fresh meat preservation to replace petrochemical polymers that are non-biodegradable and used in multilayers that are extremely difficult to recycle. Nevertheless, the lower water and oxygen barrier of PLA can still restrict its application in products where, for instance, moisture losses or oxidation during storage could be a limiting factor. Thus, high-barrier formulations and structures based on this biopolymer or others will be needed. According to these requirements, future studies will deal with the evaluation of more high-performance bioplastic films for preserving fresh foodstuffs, selecting those that are usually packaged in plastic due to their limited shelf life, whose rapid deterioration represents significant economic losses, and whose market price allows the cost of innovation to be absorbed, such as chicken breast, fish fillets, fresh pasta, or cheese.

## Figures and Tables

**Table 1:** Layer thicknesses of the polylactide (PLA), M1, and M2 multilayer films.

Film	Thickness (µm)					
	Layer 1	Layer 2	Layer 3	Layer 4	Layer 5	Total
PLA	1	18	1	-	-	20
M1	21	5	22	90	-	138
M2	5	17	5	16	60	103

**Table 2:** Total thickness and permeance to water and limonene vapors and oxygen of the polylactide (PLA), M1, and M2 multilayer films.

Film	Thicknesses (µm)	Water Vapor Permeance × 10^11^ (kg.m^−2^.Pa^−1^.s^−1^)	Limonene Permeance × 10^10^ (kg.m^−2^.Pa^−1^.s^−1^)	Oxygen Permeance × 10^14^ (m^3^.m^−2^.Pa^−1^.s^−1^)
PLA	20 ± 1 ^a^	92.59 ± 5.82 ^a^	2.20 ± 0.17 ^a^	11.54 ± 2.02 ^a^
M1	137 ± 3 ^b^	0.58 ± 0.40 ^b^	1.51 ± 0.14 ^b^	0.02 ± 0.00 ^b^
M2	98 ± 2 ^c^	2.32 ± 1.67 ^c^	0.69 ± 0.04 ^c^	0.97 ± 0.03 ^c^

^a–c^ Different superscript letters within the same column indicate significant differences among the samples (*p* < 0.05).

**Table 3:** Optical properties in terms of lightness (L*), chroma (C_ab_*), hue angle (h_ab_*), and internal transmittance (T_i_) at 550 nm of the polylactide (PLA), M1, and M2 multilayer films.

Film	L*	C_ab_*	h_ab_*	*Ti* (550 nm)
PLA	94.9 ± 1.0 ^a^	4.3 ± 1.0 ^a^	127 ± 7.0 ^a^	0.93 ± 0.004 ^a^
M1	84.6 ± 1.0 ^b^	2.6 ± 0.4 ^b^	132 ± 4.0 ^a^	0.90 ± 0.01 ^b^
M2	85.6 ± 1.0 ^b^	1.9 ± 0.2 ^c^	148 ± 1.4 ^b^	0.91 ± 0.01 ^b^

^a–c^ Different superscript letters within the same column indicate significant differences among the samples (*p* < 0.05).

**Table 4:** Development of pH and weight loss of the pork meat samples packaged in the polylactide (PLA), M1, and M2 multilayer films during storage.

Film	Storage Time (Days)			
	3	7	11	15
	pH			
Control	5.43 ± 0.01 ^a4^	5.84 ± 0.15 ^a3^	7.06 ± 0.09 ^a2^	7.66 ± 0.13 ^a1^
PLA	5.44 ± 0.02 ^a3^	5.88 ± 0.05 ^a1^	5.56 ± 0.02 ^b2^	5.25 ± 0.04 ^b4^
M1	5.41 ± 0.02 ^a1^	5.30 ± 0.01 ^b2^	5.32 ± 0.02 ^c2^	5.24 ± 0.01 ^b3^
M2	5.42 ± 0.03 ^a1^	5.33 ± 0.01 ^b2^	5.30 ± 0.01 ^c3^	5.21 ± 0.01 ^b4^
	**Weight loss (%)**			
PLA	1.930 ± 0.300 ^a3^	3.400 ± 1.160 ^a3^	8.410 ± 1.500 ^a2^	14.130 ± 1.300 ^a1^
M1	0.050 ± 0.004 ^b3^	0.020 ± 0.010 ^b4^	0.100 ± 0.010 ^b2^	1.520 ± 0.800 ^b1^
M2	0.010 ± 0.001 ^c3^	0.040 ± 0.010 ^c2^	0.170 ± 0.110 ^c1^	0.210 ± 0.100 ^c1^

Different superscripts within the same column indicate significant differences among samples for the same storage time (^a–c^) or due to storage time for the same sample (^1–4^) (*p* < 0.05).

## Data Availability

Data is contained within the article and also available on request.
